# Metabolic Strategies in Healthcare: A New Era

**DOI:** 10.14336/AD.2021.1018

**Published:** 2022-06-01

**Authors:** Matthew CL Phillips

**Affiliations:** Department of Neurology, Waikato Hospital, Hamilton, New Zealand.

**Keywords:** health, metabolism, mitochondria dysfunction, metabolic syndrome, atherosclerosis, cancer, neurodegeneration, fasting, carbohydrate-restricted diets

## Abstract

Modern healthcare systems are founded on a disease-centric paradigm, which has conferred many notable successes against infectious disorders in the past. However, today’s leading causes of death are dominated by non-infectious “lifestyle” disorders, broadly represented by the metabolic syndrome, atherosclerosis, cancer, and neurodegeneration. Our disease-centric paradigm regards these disorders as distinct disease processes, caused and driven by disease targets that must be suppressed or eliminated to clear the disease. By contrast, a health-centric paradigm recognizes the lifestyle disorders as a series of hormonal and metabolic responses to a singular, lifestyle-induced disease of mitochondria dysfunction, a disease target that must be restored to improve health, which may be defined as optimized mitochondria function. Seen from a health-centric perspective, most drugs target a response rather than the disease, whereas metabolic strategies, such as fasting and carbohydrate-restricted diets, aim to restore mitochondria function, mitigating the impetus that underlies and drives the lifestyle disorders. Substantial human evidence indicates either strategy can effectively mitigate the metabolic syndrome. Preliminary evidence also indicates potential benefits in atherosclerosis, cancer, and neurodegeneration. Given the existing evidence, integrating metabolic strategies into modern healthcare systems should be identified as a global health priority.

## 1.Introduction

During the 19th century, Louis Pasteur and Antoine Bechamp engaged in a protracted debate on the origin of infectious disease [1]. Pasteur championed germ theory, a “disease-centric” theory that emphasized pathogens, such as bacteria and viruses, as the origin of disease. Pathogens had to be targeted and eliminated to clear the disease. Bechamp, on the other hand, advocated terrain theory (also known as host theory), a “health-centric” theory that recognized most pathogens as scavengers, capable of instigating disease only in a weakened host. Terrain theory emphasized a dysfunction in the body’s own tissues and cells as the origin of disease, implicating a disease target that needed to be restored, which would in turn improve the body’s health and resistance to infections. However, the finite knowledge of the era was unable to identify a suitable restorative target, which resulted in vague mechanistic explanations and treatment recommendations. Towards the end of the century, germ theory emerged as the dominant paradigm whilst terrain theory faded into obscurity.

Since this dispute concluded, germ theory’s disease-centric paradigm has expanded to become the conceptual framework applied to most disorders encountered by our modern healthcare systems, including the non-infectious “lifestyle” disorders which dominate the leading causes of death in the west today [2]. These disorders are broadly represented by four processes - the metabolic syndrome (a major predisposing factor to most leading causes of death, including COVID-19) [3], atherosclerosis, cancer, and neurodegeneration. The disease-centric paradigm regards each of these disorders as a disease process itself, caused and driven by pathogenic targets that must be suppressed or eliminated, typically with drugs. The metabolic syndrome, for example, is regarded as a constellation of disease markers caused by glucose intolerance and/or insulin resistance, so blood glucose levels and insulin resistance are targeted [4]. Atherosclerosis is regarded as a disease primarily caused by a high blood LDL cholesterol [5], so cholesterol is targeted [6]. Cancer is regarded as a disease caused by nuclear mutations [7], so the mutations are targeted [8]. Neurodegeneration is regarded as a disease caused by abnormal protein aggregates [9,10], so the aggregates are targeted [11]. Despite prodigious funding and a multitude of drugs, the disease-centric paradigm has not halted the rise of these disorders. Rather than investing further in this approach, perhaps we should reconsider the paradigm.

Given today’s knowledge, a health-centric paradigm can be elaborated based on a comprehensive body of research identifying mitochondria dysfunction as a common process underlying the metabolic syndrome [12], atherosclerosis [13,14], cancer [15,16], and neurodegeneration [17,18]. Our disease-centric paradigm continues to regard these disorders as distinct disease processes, caused and driven by disease targets that must be suppressed or eliminated to clear the disease. By contrast, a health-centric paradigm recognizes the lifestyle disorders as a series of hormonal and metabolic responses to a singular, lifestyle-induced disease of mitochondria dysfunction, a disease target that must be restored to improve health, which may be defined as optimized mitochondria function. Seen from a health-centric perspective, most drugs aim to suppress or eliminate a response rather than address the actual disease, whereas metabolic strategies, such as fasting and carbohydrate-restricted diets, aim to restore mitochondria function, mitigating the impetus that underlies and drives the lifestyle disorders. To understand why a health-centric perspective may be applicable to the lifestyle disorders, some evolutionary context is required.

## 2.A Transition in Dietary Lifestyle

**Figure 1.**
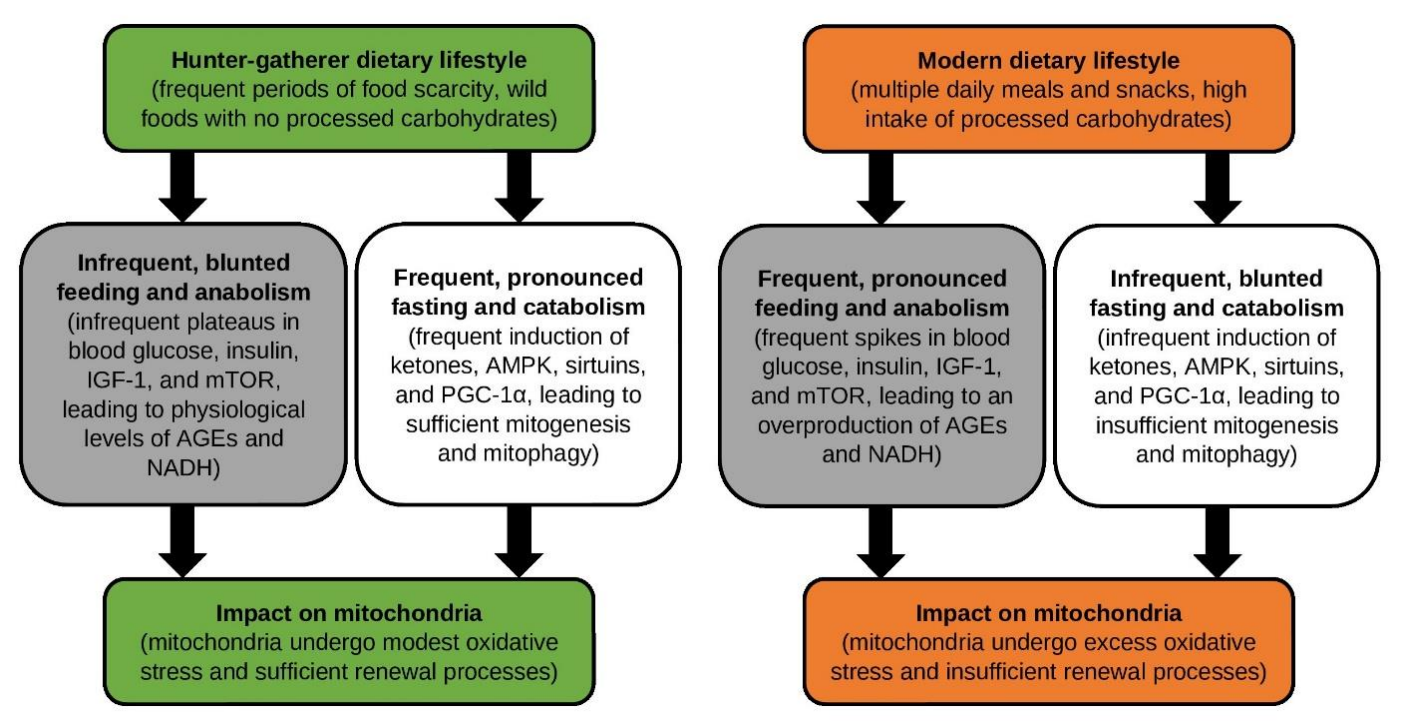
Comparing the impact of hunter-gatherer versus modern dietary lifestyles on metabolism and mitochondria function in humans.

The rapid rise in prevalence of the lifestyle disorders in recent decades implicates changes in environmental factors such as diet, sleep, exercise, workload, and social interaction; arguably, the most profound changes have occurred in the frequency and content of the human diet [19] (Fig. 1). For 2-3 million years, humans adhered to a hunter-gatherer dietary lifestyle characterized by frequent periods of food scarcity and wild (pre-agrarian, unprocessed) foods [20]. By comparison, the modern dietary lifestyle, which is characterized by multiple daily meals and snacks with a high intake of processed carbohydrates, has existed in its current form for a comparatively fleeting 50 years or so [19]. Clearly, there has been insufficient evolutionary time for human metabolism to adapt to such a dramatic change in dietary lifestyle.

### 2.1 The Hunter-Gatherer Dietary Lifestyle

Metabolically, the hunter-gatherer dietary lifestyle fostered a balanced oscillation between two states - anabolism, which emphasized growth and replication, and catabolism, which emphasized maintenance and repair. Hunter-gatherer meals consisted of wild animal and plant products (ranging from primarily carnivorous to largely vegetarian, depending on geography), leading to a digestible carbohydrate load that was either very low or, if high, accompanied by ample fiber, which slowed carbohydrate absorption and was fermented by colon bacteria to generate short-chain fatty acids for epithelial cells [20]. Postprandially, such an eating pattern induced plateau-like, finite elevations in blood glucose and nutrient sensors such as insulin, insulin-like growth factor 1 (IGF-1), and mammalian target of rapamycin (mTOR), promoting tempered growth. Intracellularly, the limited rise in glycolysis generated relatively low, physiological levels of reduced NADH (and FADH2) intermediates and electron flow along the mitochondria respiratory chain, leading to modest levels of reactive oxygen species (ROS) and oxidative stress [21,22]. The slight increase in ROS would have also encouraged mitochondria hormesis, fostering long-term decreases in oxidative stress [23]. Moreover, hunter-gatherers endured frequent periods of food scarcity, which would have routinely forced the body to “switch” from glucose to fat-derived ketones as the primary energy source for cells and mitochondria [24]. Compared with glucose, ketones generated more energy yet fewer ROS per unit of oxygen [25], maintained signalling functions aimed at controlling oxidative stress and inflammation [19], and may have been metabolized as a partial substitute for the functions of fiber [26]. Beyond ketones, the periods of food scarcity inherent to a hunter-gatherer lifestyle would have induced the key energy sensor AMP-activated protein kinase (AMPK) to stimulate autophagy, an intracellular pathway aimed at removing and recycling oxidatively damaged proteins and mitochondria [27]. The induction of AMPK (and sirtuins) additionally upregulated the expression of peroxisome proliferator-activated receptor-γ coactivator-1α (PGC-1α), inducing biogenesis within the body’s pool of mitochondria [27]. Importantly, the repeated periods of food scarcity and catabolism positioned cells and mitochondria for an enhanced recovery phase during subsequent periods of refeeding and anabolism.

### 2.2 The Modern Dietary Lifestyle

The modern dietary lifestyle induces frequent, pronounced periods of anabolism and infrequent, blunted periods of catabolism. Most humans today consume multiple daily meals and snacks with a high intake of processed carbohydrates (particularly sugar), leading to frequent glucose (and likely fructose) “spikes” as well as heightened elevations in insulin, IGF-1, and mTOR [19], provoking exaggerated growth. Intracellularly, the amplified stimulation of glycolysis leads to an overproduction of unstable advanced glycation end products (AGEs) [28,29], with a potentially disproportionate contribution from fructose as it is ten times more reactive than glucose [30]. The abnormal stimulation of glycolysis also overproduces NADH (and FADH2) intermediates, leading to electron “overflow” along the mitochondria respiratory chain [21,22]. The combined overproduction of AGEs and NADH culminates in excess ROS and damage to intracellular components. Mitochondria are particularly vulnerable to damage owing to their comparative deficiency in repair mechanisms [31]. Moreover, given that most societies no longer endure frequent periods of food scarcity, modern humans undergo insufficient ketogenic metabolic switching, AMPK-induced mitophagy, and mitogenesis [24]. Ultimately, the exorbitant emphasis on anabolism over catabolism places the body’s mitochondria in a tenuous position, exposed to excess oxidative stress yet unable to sufficiently renew, setting the stage for mitochondria dysfunction.

## 3.Mitochondria Dysfunction: A Singular Disease

**Figure 2.**
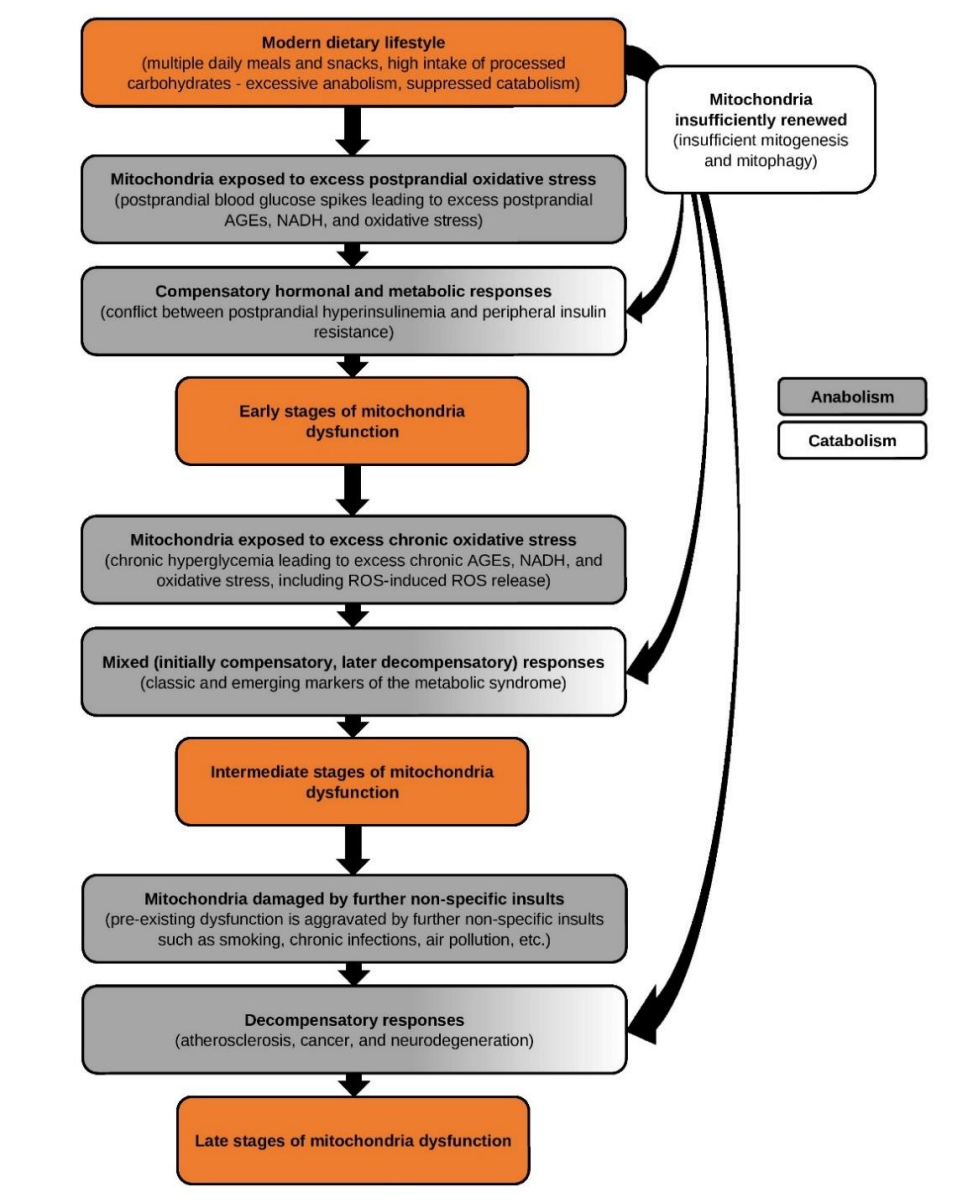
Health-centric paradigm portraying the lifestyle disorders as a series of hormonal and metabolic responses to a singular, lifestyle-induced disease of mitochondria dysfunction.

Seen from an evolutionary and health-centric perspective, the primary factor driving mitochondria dysfunction today is the modern dietary lifestyle, which induces excessive anabolism and suppresses catabolism, exposing mitochondria to excess oxidative stress and insufficient renewal processes (Fig. 2). If this lifestyle persists, the body initiates a series of compensatory hormonal and metabolic responses aimed at mitigating mitochondria exposure to oxidative stress, most conspicuously represented by postprandial hyperinsulinemia, peripheral insulin resistance, and several markers of the metabolic syndrome. Over time, these compensatory responses fail to prevent mitochondria dysfunction, leading to decompensatory responses in the guise of atherosclerosis, cancer, and neurodegeneration.

### 3.1 Conflict Between Hyperinsulinemia and Insulin Resistance

Repeated mitochondria exposure to excess postprandial blood glucose spikes, AGEs, NADH, and the ensuing oxidative stress triggers an escalating conflict between postprandial hyperinsulinemia and peripheral insulin resistance. Although generally regarded as a disease process arising from post-receptor defects in insulin signalling [32], peripheral insulin resistance can alternatively be recognized as a compensatory response resulting from downregulated (not defective) cell insulin signalling, which aims to prevent mitochondria exposure to excess ROS. To compensate for the spikes, the pancreatic β-cells secrete additional insulin to redistribute excess blood glucose into adipose and muscle tissue, with the latter responsible for 80-85% of glucose disposal [33]. However, to prevent a corresponding excess of intracellular glucose, adipose and muscle cells compensate by progressively downregulating their insulin responsivity. In turn, the pancreas increases its β-cell mass, leading to compensatory hyperinsulinemia [32], which elicits further insulin resistance and so on, culminating in years or decades of an escalating conflict between postprandial hyperinsulinemia and insulin resistance [34]. Each of these compensatory responses attempts to redistribute the harmful effects of excess dietary glucose away from a specific body compartment, but an increase in one provokes an increase in the other such that mitochondria are inevitably exposed to excess postprandial oxidative stress, leading to the early stages of dysfunction.

### 3.2 The Metabolic Syndrome

Once mitochondria dysfunction has been established, mitochondria exposure to excess chronic hyperglycemia, AGEs, NADH, and oxidative stress is also accompanied by a vicious cycle of “ROS-induced ROS release” in which chronically amplified ROS production induces further dysfunction, and vice versa [35], contributing to a cluster of markers collectively known as the metabolic syndrome. Classically, the syndrome is defined by five markers - a high fasting blood glucose, high blood triglycerides, low blood high-density lipoprotein (HDL), abdominal obesity, and hypertension [4]. However, emerging markers of the syndrome include non-alcoholic fatty liver disease (NAFLD) [36], a high ratio of small dense to large buoyant low-density lipoprotein (LDL) particles [37], chronic systemic inflammation [38], and (potentially) brain insulin resistance [39]. Although generally regarded as a series of disease processes attributable to peripheral insulin resistance [4], the metabolic syndrome can alternatively be recognized as a series of compensatory responses broadly aimed at preventing mitochondria exposure to excess ROS, many of which later decompensate such that they ultimately exacerbate ROS production. Typically, the first marker to appear is a high fasting blood glucose, or prediabetes, which reflects failed compensatory hyperinsulinemia in the face of increasing peripheral insulin resistance. Further decompensation leads to type 2 diabetes, with a yearly conversion rate of 5-10% [40]. Given the body’s limited ability to store glucose, the liver compensates for chronic hyperglycemia by converting excess dietary glucose into triglycerides, which it can store, leading to fatty liver and NAFLD [33,36], or export to the circulation, leading to a dyslipidemic blood lipid profile characterized by high triglycerides, a low HDL cholesterol, and an increased ratio of small dense to large buoyant LDL particles [4,37]. The body increases its deposition of peripheral subcutaneous fat to compensate for the triglyceride load, but if these reserves are exceeded, maladaptive visceral fat is deposited within and around the intraperitoneal organs, leading to abdominal obesity [4]. Deposition of this “sick fat” is associated with an aberrant adipokine profile [41], a major contributor to chronic systemic inflammation [38]. Chronic exposure of endothelial cells to elevated blood glucose levels leads to the initial stages of mitochondria dysfunction in endothelial cells, with the resulting endothelial dysfunction contributing to impaired vasodilation and hypertension [13,42]. Neurons may initially respond to excess oxidative stress and mitochondria dysfunction by downregulating insulin signalling, which contributes to brain insulin resistance [39]. The eventual failure of the metabolic syndrome to protect mitochondria from excess chronic oxidative stress leads to the intermediate stages of dysfunction.

### 3.3 Atherosclerosis

Atherosclerosis is characterized by an accumulation of oxidized LDL cholesterol, inflammatory cells, and cell debris within the arterial intima [5]. For over 50 years, the cholesterol theory has regarded atherosclerosis as a disease caused by a high blood LDL cholesterol [43], which triggers an accumulation of oxidized LDL cholesterol in the intima leading to endothelial dysfunction, chronic arterial inflammation, immune activation, and plaque formation, culminating in cardiovascular events such as myocardial infarction and ischemic stroke.

The cholesterol theory of atherosclerosis bears several shortcomings. First, it does not sufficiently address the striking oxidative damage and mitochondria dysfunction observed in human plaques [13,14]. Second, many non-specific insults can precipitate atherosclerosis including smoking, chronic infections, and air pollution, amongst other factors [44]. The cholesterol theory does not explain how such a specific process can be precipitated by a variety of non-specific insults. Third, although many observational studies have correlated a higher blood LDL cholesterol with worsened atherosclerosis and increased cardiovascular events [45], such a correlation does not permit the inference that LDL cholesterol causes atherosclerosis. Moreover, some very large studies have found the opposite correlation. For example, a cohort study involving nearly 140,000 individuals hospitalized with coronary artery disease revealed that half of them had a below-normal blood LDL cholesterol [46]. Conversely, a recent systematic review involving 68,000 individuals 60 years of age or older showed a high blood LDL cholesterol was associated with increased (rather than decreased) longevity [47]. Fourth, although reviews of randomized trials have suggested reducing blood LDL cholesterol lowers cardiovascular events [45,48,49], the conclusions of these reviews are debatable given the existence of financial conflicts, the exclusion of negative trials from the analyses, and the possibility that the putative benefits of LDL-lowering were overemphasized [50].

Under a health-centric paradigm, atherosclerosis can be recognized as a series of decompensatory responses to worsening mitochondria dysfunction in endothelial cells. Once established, mitochondria dysfunction is further aggravated by endothelial exposure to multiple blood-borne factors which all share a capacity to oxidatively damage mitochondria, including smoking [51], chronic infections [52], and air pollution [53], explaining how various non-specific insults can precipitate a common, specific process. Oxidative damage to endothelial mitochondria leads to endothelial dysfunction, followed by the accumulation of oxidized LDL cholesterol in the intima and latter stages of atherosclerosis. Importantly, these events generally occur in the context of one or more markers of the metabolic syndrome, particularly a dyslipidemic blood lipid profile, which frequently is accompanied by a high blood LDL cholesterol. Yet it is also crucial to realize that the dyslipidemia and the atherosclerotic process both represent responses to a single, underlying etiology of mitochondria dysfunction, explaining why many (though not all) studies have repeatedly linked a high blood LDL cholesterol with the cardiovascular sequelae of atherosclerosis. Seen this way, the actual “role” of oxidized LDL in the intima is unclear - although it may play a contributory role to endothelial dysfunction and atherosclerosis, it may alternatively play a passive role, or even a protective one [54]. Regardless of any putative role, oxidized LDL can be viewed as an intermediary effect of atherosclerosis, not the cause, which explains the questionable ability of cholesterol-lowering drugs to decrease cardiovascular events.

### 3.4 Cancer

Cancer is characterized by the acquisition of a series of hallmark properties in cells followed by abnormal cell growth and spread throughout the body [7,55]. Over 100 years have passed since the inception of the somatic mutation theory [56], which continues to regard cancer as a disease caused by nuclear mutations leading to activated oncogenes, inactivated tumour-suppressor genes, and malignant tumour growth.

The somatic mutation theory contains numerous inconsistencies with respect to sporadic cancers, which represent over 95% of cancers. First, it does not explain the abundant evidence indicating all types of human cancers express high levels of ROS and mitochondrial abnormalities, including reduced numbers, defective cardiolipin, disorganized cristae, and fusion-fission dysequilibrium, culminating in dysfunction [15,16]. Although several investigators have pointed out that some tumour cells display normal oxygen consumption, suggesting normal respiration [57], tumour mitochondria also show higher rates of mitochondrial uncoupling (potentially as a means of dampening oxidative damage) [58], indicating deficient respiration despite normal oxygen consumption. Second, many non-specific insults can precipitate cancer including smoking, chemical carcinogens, and radiation, amongst others, leading to an “oncogenic paradox” [59]. Cancer is a highly specific process, involving specific changes in specific chemical machinery, so its precipitation by a plethora of non-specific factors is paradoxical. Third, somatic mutation theory is inconsistent with the results of cytoplasm transfer experiments, which have been confirmed by many investigators [60]. If nuclear mutations are the origin of cancer, combining a cancer cell nucleus with normal mitochondria should maintain the cancer state, yet the normal state persists. Conversely, combining a normal nucleus with cancer cell mitochondria should maintain the normal state, yet the cancer state persists. These findings suggest malignant transformation is dictated by mitochondria, not the nucleus. Fourth, somatic mutation theory does not sufficiently address the Warburg Effect, which refers to a highly upregulated aerobic fermentation of glucose (and, in some tumours, glutamine) that is seen as a near-universal feature of malignant tumours [61]. Warburg considered this metabolic alteration to be an inheritance from less differentiated ancestors in Earth’s past for which the default cell state was fermentation-driven unbridled proliferation. Fifth, somatic mutation theory does not explain the vast diversity of nuclear mutations in cancer, with a single human tumour exhibiting anywhere from zero to several hundred non-synonymous mutations [62]. The fact that up to 35% of tumours display zero mutations is difficult to reconcile with the notion that mutations cause cancer [63].

Under a health-centric paradigm, cancer can be recognized as a series of decompensatory responses to worsening mitochondria dysfunction. Established dysfunction is further aggravated by smoking, chemical carcinogens, radiation, and other factors which all share a capacity to oxidatively damage mitochondria, resolving the oncogenic paradox [16]. In the setting of worsening dysfunction and a potential cell energy shortage, mitochondria invoke a retrograde stress response characterized by a series of nuclear genetic and epigenetic alterations aimed at upregulating glycolytic pathways [64], allowing the cell to partially compensate for damaged respiration by resorting to its default state of fermentation-driven unbridled proliferation [61]. The retrograde stress response places mitochondria as the key determinants of malignant transformation, consistent with the results of cytoplasm transfer experiments. The cell transition to aerobic fermentation occurs over a long period of time, with weakly fermenting cells perishing and strong fermenters surviving [61], explaining the high prevalence of the Warburg Effect in growing tumours. Despite compensation by the Warburg Effect, DNA repair enzyme function still requires normal respiration and is therefore impaired, leading to an accumulation of random nuclear mutations, followed by the remaining cancer hallmarks [16]. Irrespective of whether nuclear mutations are semi-purposefully evoked by the retrograde stress response and/or randomly appear in the setting of impaired repair processes, both can be viewed as intermediary effects of cancer, not the cause, which explains the diversity of non-synonymous nuclear mutations seen in human tumours.

### 3.5 Neurodegeneration

The most common neurodegenerative disorder is Alzheimer’s disease (AD), which is characterized by amyloid plaques (containing mostly amyloid-β) [9], neurofibrillary tangles (containing hyperphosphorylated tau) [10], and massive neuron loss. For nearly 30 years, the amyloid cascade theory (as well as the more recent tau theory) has regarded AD as a disease caused by neurotoxic protein aggregates, which trigger a cascade of events leading to chronic neuroinflammation, neuron loss, cognitive impairment, and dementia.

The amyloid and tau cascade theories bear several shortcomings with respect to sporadic AD, which represents over 90% of AD. First, neither theory explains the prominent oxidative damage and mitochondria dysfunction observed in AD neurons (which are the earliest events in AD, preceding plaques and tangles), with mitochondria displaying reduced numbers, abnormal shapes, fusion-fission dysequilibrium, and deficient respiratory chain enzymes [17,18]. Second, many non-specific insults can precipitate AD including aluminum, pesticides, and air pollution, amongst other factors [65]. The amyloid and tau cascade theories do not explain how such a specific process can be precipitated by a variety of non-specific insults. Third, if either theory is correct, the extent of the plaque or tangle load should correlate with the degree of neuron loss and cognitive impairment; however, the correlation is weak. For example, 20-40% of non-demented elderly individuals have plaque loads meeting the criteria for AD, with nearly all of them showing tangles [66]. Conversely, neurodegeneration can occur in the absence of plaques [67]. Fourth, many randomized trials have indicated drugs targeting amyloid-β do not improve the cognitive impairment, even when plaques are successfully removed [68].

Under a health-centric paradigm, AD can be recognized as a series of decompensatory responses to worsening mitochondria dysfunction in neurons. Established dysfunction is further aggravated by neuron exposure to multiple factors which all share a capacity to oxidatively damage mitochondria, including aluminum [69], pesticides [70], and air pollution [71], explaining how various non-specific insults can precipitate a common, specific process. Worsening mitochondria dysfunction forces neurons to walk a thin line, faced with a cell energy shortage on one end and escalating oxidative stress on the other. Unlike most cells, energy-demanding neurons have a limited ability to upregulate their glycolytic capacity when respiration is damaged [72], therefore it is not viable for them to transition to aerobic fermentation and the Warburg Effect, which results in an imminent energy shortage. Yet given their ongoing heavy reliance on oxygen and relative dearth of antioxidants, neurons also face increasing oxidative damage to their intracellular components [73]. Under these conditions, it becomes bioenergetically onerous for neurons to degrade damaged, potentially neurotoxic proteins, leading to a compensatory re-routing of metabolic priorities and an increased generation of plaques and tangles [73]. Seen this way, the “role” of plaques and tangles is unclear - although they may exacerbate the AD process, they may alternatively play a passive role, or even a protective one by sequestering neurotoxic proteins into comparatively inert aggregates [73]. Regardless of any putative role, plaques and tangles can be viewed as intermediary effects of AD, not the cause, which explains the weak correlation between plaque or tangle load with the degree of neuron loss and cognitive impairment, as well as the inability of drugs targeting amyloid-β to improve the cognitive impairment.

## 4.Metabolic Strategies: Restoring Mitochondria Function

**Figure 3.**
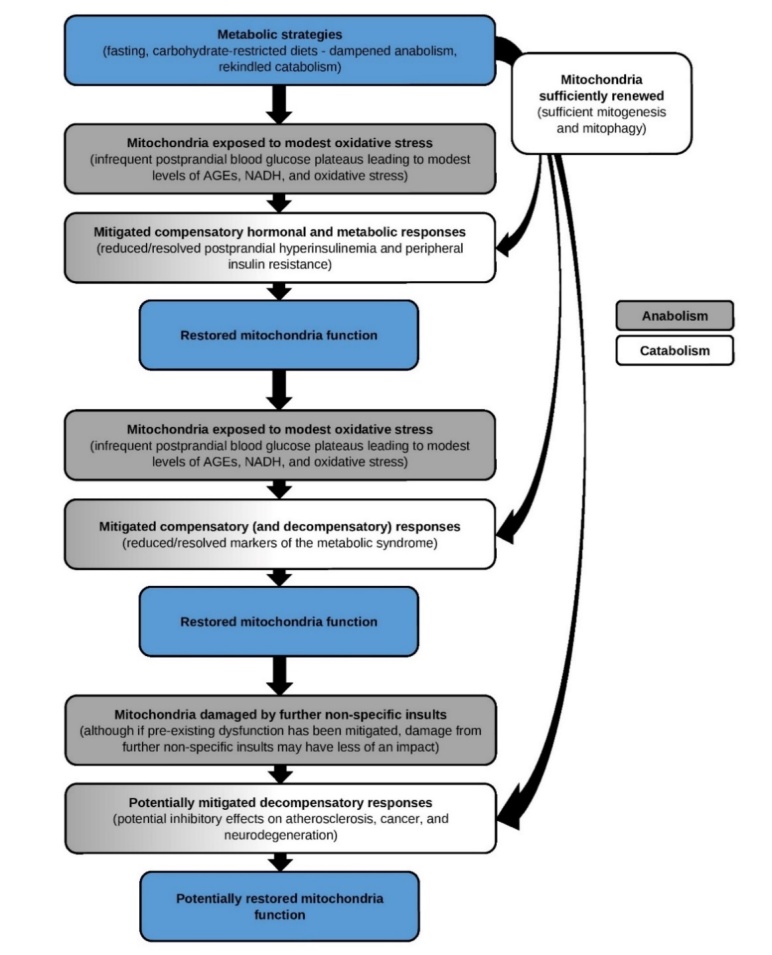
Health-centric paradigm portraying the impact of metabolic strategies on metabolism, mitochondria function, and the lifestyle disorders.

Seen from a health-centric perspective, metabolic strategies aim to restore mitochondria function by dampening anabolism and rekindling catabolic processes, which reduces mitochondria exposure to oxidative stress and enhances renewal of the mitochondria pool, mitigating the impetus that underlies and drives the lifestyle disorders (Fig. 3). Although a variety of metabolic strategies exist including fasting, carbohydrate-restricted diets, calorie restriction, and specific forms of exercise, all of which have the capacity to restore mitochondria function, the two most applicable and impactful strategies are fasting and carbohydrate-restricted diets, which induce similar mechanisms. Dozens of human interventional trials have indicated either strategy can effectively mitigate the metabolic syndrome. Preliminary studies have also indicated potential benefits in atherosclerosis, cancer, and neurodegeneration.

### 4.1 Fasting

Fasting is a voluntary abstinence from food and drink that permits water, calorie-free fluids, or limited calorie-restricted meals for specified periods of time [74] (Table 1). “Intermittent” fasting involves fasts with a duration of 12-48 hours and may be subdivided into time-restricted feeding (TRF), alternate daily fasting (ADF), and 5:2 fasting regimens. “Periodic” fasting involves fasts of 2-21 days and may be subdivided into prolonged fasting and fasting-mimicking diet (FMD) regimens. The essential aim of fasting is to establish a metabolic balance by creating frequent, pronounced periods of catabolism, generating an orchestra of metabolic alterations at the systemic, cellular, and mitochondrial level that restore mitochondria function [75]. Systemically, fasting decreases blood glucose, insulin, and IGF-1 and increases ketones and antioxidants, diminishing mitochondria oxidative stress and encouraging mitochondria hormesis. Intracellularly, fasting decreases mTOR and increases AMPK, sirtuins, and PGC-1α, enhancing mitochondria renewal processes.

Many human interventional studies have shown fasting improves hyperinsulinemia, insulin resistance, and virtually all markers of the metabolic syndrome. Fasting blood insulin levels are reduced by 20-40% with TRF [76,77] and by 50-60% with ADF [78,79]. Older studies have demonstrated prolonged fasting frequently resolves insulin resistance and type 2 diabetes, which includes a reduction or cessation of diabetes medications [80,81]. More recent studies, most of which are randomized controlled trials, have also revealed substantial reductions in insulin resistance after several weeks to months of TRF [76,77,82], ADF [78], or 5:2 fasting [83-85]. Body weight is reduced by 3-9% in overweight or obese individuals undergoing 8-24 weeks of TRF [77,82,86], ADF [78,87-89,90,91], 5:2 fasting [83-85], or an FMD [92], and by 4% after a 5-day prolonged fast [93]. Importantly, the weight loss targets visceral fat, spares lean mass (particularly when combined with exercise) [87,91], and persists for at least 1 year [78,86,90]. Fasting improves the blood lipid profile in diabetic, overweight, and obese individuals, lowering triglycerides and increasing LDL particle size after 8-24 weeks of TRF [77], ADF [87-89,91], or 5:2 fasting [83]. Both 5:2 fasting and FMDs reduce liver fat, improving NAFLD [85,94]. Blood pressure in hypertensive individuals is reduced after 12 weeks of TRF (by 5 mmHg systolic, 7 mmHg diastolic) [77] and after a single prolonged fast averaging 10 days in duration (by 7-17 mmHg systolic, 5-9 mmHg diastolic), with the latter allowing 44% of individuals to reduce their anti-hypertensive medications and 24% to cease them [95]. Markers of systemic inflammation such as tumour necrosis factor alpha (TNF-α), interleukin 6 (IL-6), and C-reactive protein (CRP) are reduced after 8-24 weeks of TRF [96], ADF [91], 5:2 fasting [83,84], or an FMD [92].

**Table 1:** Evidence for fasting regimens in the lifestyle disorders (using only data from human interventional studies).

	Fasting regimen	Definition	Evidence in lifestyle disorders (using only data from human interventional studies)
Intermittent fasting	Time-restricted feeding (TRF)	Fasting 12-20 hours daily, with a 4-12-hour ad libitum feeding window	Reduces fasting blood insulin levels by 20-40% Reduces insulin resistance Induces 3-9% weight loss over 8-24 weeks Lowers triglycerides and increases LDL particle size Modestly decreases hypertension Suppresses inflammation Improves adipokine profile Alleviates adverse effects of chemotherapy
	Alternate daily fasting (ADF)	Fasting every other day, with ad libitum feeding on non-fasted days	Reduces fasting blood insulin levels by 50-60% Reduces insulin resistance Induces 3-9% weight loss over 8-24 weeks Lowers triglycerides and increases LDL particle size Suppresses inflammation Improves adipokine profile
	5:2 fasting	Fasting 2 consecutive days per week, with a 5-day ad libitum feeding period	Reduces insulin resistance Induces 3-9% weight loss over 8-24 weeks Lowers triglycerides and increases LDL particle size Reduces liver fat in NAFLD Suppresses inflammation Improves adipokine profile
Periodic fasting	Prolonged fasting	Fasting 2-21 consecutive days, followed by at least 7 ad libitum feeding days	Resolves insulin resistance, including type 2 diabetes Induces 4-11% weight loss over 4-21 days Lowers triglycerides and increases LDL particle size Substantially decreases hypertension Improves quality of life during chemotherapy Alleviates adverse effects of chemotherapy Enhances resistance of normal cells to chemotherapy
	Fasting-mimicking diet (FMD)	Fasting (30-50% calorie restriction) 4-7 consecutive days, followed by 10-25 ad libitum feeding days	Reduces insulin resistance Induces 3-4% weight loss over 3 months Reduces liver fat in NAFLD Suppresses inflammation

Beyond restoring mitochondria function and mitigating the metabolic syndrome, fasting may inhibit atherosclerosis by augmenting endothelial function, suppressing chronic arterial inflammation, and improving the adipokine profile [97,98]. No animal or human interventional studies have yet investigated the direct impact of fasting on atherosclerosis. However, animal studies have shown fasting mitigates the damage induced by cardiovascular events, reducing myocardial infarct size by 35% [99] and ischemic stroke size by 50% [100]. It is also worth considering human observational studies involving calorie restriction, with one study showing long-term calorie-restricted individuals displayed a 40% reduction in carotid artery intima-media thickness, no ultrasound evidence of plaques, and improved heart function compared with individuals on a modern diet [101]. Moreover, randomized crossover trials in humans have demonstrated ketone infusions induce a striking 75% increase in myocardial blood flow and a 40% increase in cardiac output compared with saline fusions [102,103].

Beyond restoring mitochondria function, fasting may inhibit tumour growth by eliciting differential stress resistance and sensitization (enhancing resistance in normal cells while sensitizing cancer cells to multiple stressors), generating ketones (which are not effectively utilized by cancer cells as an energy source), downregulating growth factors, restricting the availability of fermentable fuels, inducing epigenetic alterations that do not favour the malignant phenotype, and activating antitumour immunity [104]. In animals, fasting and calorie restriction reduce tumour incidence by roughly 75% [105]. In humans, six interventional studies (including three randomized trials) have explored the impact of fasting alongside standard treatments in a variety of advanced cancers [106-111]. Collectively, these studies reveal fasting improves quality of life, alleviates adverse effects, and enhances stress resistance in normal cells during chemotherapy. Notably, although most of these studies involved prolonged fasts, the fasts were still relatively short (the study with the longest fasts averaged roughly 90 hours per fast) [110]. Longer fasts in cancer patients are rare, although there have been two case reports of patients with advanced cancer undergoing fasts of 7-21 days in duration, both of whom showed long-term (and ongoing) tumour regression and survival in the absence of any standard cancer treatments [112,113].

Beyond restoring mitochondria function, fasting may ameliorate neurodegeneration by generating ketones (a superior energy source for neurons that generates fewer ROS, circumvents brain insulin resistance, and increases the expression of neurotrophic factors), suppressing chronic neuroinflammation, and stimulating autophagy [75,114]. Fasting generally slows neurodegeneration and improves functional outcomes in animal models of AD and Parkinson’s disease (PD) [115]. No interventional studies have yet investigated the impact of fasting on neurodegeneration in humans. However, numerous studies have shown fasting improves cognition, energy, mood, sleep, self-confidence, and quality of life in non-demented individuals [83,84,86,93], which would more than likely be beneficial in individuals with AD or PD. It is also worth considering interventional studies involving calorie restriction, with one recent randomized controlled trial showing obese, cognitively impaired, calorie-restricted individuals developed improved memory, executive function, and global cognition compared with individuals on an ad libitum diet [116].

### 4.2 Carbohydrate-Restricted Diets

Carbohydrate-restricted diets limit the daily intake of digestible carbohydrate, which is replaced with one or more of fat, protein, or fiber (ideally, fat) [34] (Table 2). Low-carbohydrate diet (LCD) regimens typically limit carbohydrate intake to 50-100 g daily. Ketogenic diet (KD) regimens restrict carbohydrate intake to below 50 g daily, which leads to the induction of physiological ketosis (indicated by a blood beta-hydroxybutyrate level of 0.5-0.6 mmol/L or higher). KDs may be primarily carnivorous, vegetarian, or omnivorous and modified to appeal to any ethnic preference, so long as physiological ketosis is achieved. The essential aim of carbohydrate-restricted diets is to blunt the metabolic impact of feeding and partially activate the fasting-elicited orchestra of mechanisms that restore mitochondria function [117]. Systemically, LCDs and KDs decrease blood glucose, insulin, and IGF-1, diminishing mitochondria oxidative stress and encouraging mitochondria hormesis, with KDs also inducing significant ketogenesis. Intracellularly, KDs additionally increase AMPK, sirtuins, and PGC-1α, enhancing mitochondria renewal processes.

Many human interventional studies have shown carbohydrate-restricted diets improve hyperinsulinemia, insulin resistance, and virtually all markers of the metabolic syndrome. In most cases, LCDs and KDs reduce fasting blood insulin levels by 20-40% [118]. Multiple systematic reviews of randomized controlled trials have generally demonstrated a beneficial impact of LCDs and KDs on insulin resistance [119-122]. Systematic reviews involving overweight and obese individuals also show LCDs and KDs induce more weight loss compared with low-fat diets [119,123-125]. Despite these positive findings, it is important to note many trials were limited by inadequate support for the dietary change, impacting long-term adherence. Adherence can be greatly improved using patient support programs - even a basic support program focused on educating individuals about LCDs can lead to substantial reductions in insulin resistance and 8-9% weight loss, with both changes persisting for at least 23 months [126]. With respect to KDs, a more comprehensive support program leads to the resolution of diabetes in 54% of individuals, a reduction or cessation of diabetes medications in most individuals, and an average body weight reduction of roughly 10%, with all changes maintained after 2 years [127]. Contrary to popular belief, systematic reviews of randomized controlled trials involving diabetic, overweight, and obese individuals have suggested LCDs and KDs improve the blood lipid profile by lowering triglycerides, increasing HDL cholesterol, and increasing the ratio of large buoyant to small dense LDL particles [120,121,123,128]. Both LCDs and KDs improve NAFLD (particularly KDs, which elicit a 44% reduction in liver fat after 2 weeks) [85,129,130]. Both LCDs and KDs reduce blood pressure in hypertensive individuals (by 5-11 mmHg systolic, 3-5 mmHg diastolic) for at least 1-2 years, allowing some patients to reduce or cease their anti-hypertensive medications [126,131-133]. Markers of systemic inflammation such as TNF-α, IL-6, and CRP are reduced after 3-12 months on a KD [131,134].

**Table 2:** Evidence for carbohydrate-restricted diet regimens in the lifestyle disorders (using only data from human interventional studies).

Diet regimen	Definition	Evidence in lifestyle disorders (Using only data from human interventional studies)
Low-carbohydrate diet (LCD)	Limits digestible carbohydrate intake to 50-100 g daily	Reduces fasting blood insulin levels by 20-40% Reduces insulin resistance Induces 8-9% weight loss over 1-2 years Lowers triglycerides, increases HDL, and increases LDL particle size Reduces liver fat in NAFLD Decreases hypertension
Ketogenic diet (KD)	Limits digestible carbohydrate intake to below 50 g daily	Reduces fasting blood insulin levels by 20-40% Reduces or resolves insulin resistance, including type 2 diabetes Induces 10% weight loss over 1-2 years Lowers triglycerides, increases HDL, and increases LDL particle size Substantially reduces liver fat in NAFLD Decreases hypertension Suppresses inflammation Lowers predicted risk of atherosclerotic cardiovascular events Improves quality of life in cancer Improves cognition, daily function, and quality of life in AD Improves multiple nonmotor symptoms in PD

Beyond restoring mitochondria function and mitigating the metabolic syndrome, carbohydrate-restricted diets may inhibit atherosclerosis in a similar manner to fasting by augmenting endothelial function and suppressing chronic arterial inflammation [98]. Recently, a 12-week KD was shown to decrease the aortic plaque burden in an animal model of atherosclerosis [135]. Although no human interventional studies have yet investigated the direct impact of LCDs or KDs on atherosclerosis, one systematic review of randomized controlled trials in humans calculated an atherosclerotic cardiovascular disease “risk score,” which revealed LCDs lower the predicted risk of cardiovascular events, suggesting an atheroprotective effect [124]. Moreover, a recent 18-month randomized controlled trial involving type 2 diabetics showed an LCD induced a non-significant decrease in carotid media thickness (*P* = 0.08) compared with a traditional low-fat diet [132].

Beyond restoring mitochondria function, KDs may inhibit tumour growth in a similar manner to fasting by generating ketones, downregulating growth factors, limiting fermentable fuels, inducing epigenetic alterations, and activating anti-tumour immunity [136]. Many animal studies have indicated KDs delay tumour initiation, slow tumour growth, reverse cancer-induced cachexia, and prolong survival [136]. In humans, approximately 30 interventional studies (one randomized controlled trial, the rest consisting of single-arm studies and case reports) have explored the impact of KDs, either as a stand-alone therapy or alongside standard treatments, for a variety of cancers [136]. Collectively, these studies demonstrate KDs improve quality of life, may prolong survival, and do not induce serious adverse events in cancer patients. Moreover, although KDs induce weight loss in most cancer patients, they target fat mass and preserve lean mass. Importantly, KDs appear to induce weight gain (not loss) in cachectic patients.

Beyond restoring mitochondria function, KDs may ameliorate neurodegeneration in a similar manner to fasting by generating ketones, suppressing chronic neuroinflammation, and stimulating autophagy [114,137]. KDs generally slow neurodegeneration and improve functional outcomes in animal models of AD and PD [138]. In humans, six interventional studies (including four randomized trials) have investigated the impact of KDs in AD [139-141] and PD [142-144]. The conclusions from these trials indicate KDs may improve cognition, daily function, and quality of life in individuals with AD. KDs also improve many of the nonmotor symptoms in PD, particularly those related to urinary problems, pain, fatigue, daytime sleepiness, and cognitive impairment.

## 5.Integrating Metabolic Strategies into Healthcare

Despite the existing evidence supporting the therapeutic efficacy of metabolic strategies in the lifestyle disorders, there are additional factors influencing their potential integration into modern healthcare systems. Several advantages facilitating these strategies include their adaptability to various lifestyles, freedom from long-term adverse effects, and cost-effectiveness for patients and healthcare systems. However, several key challenges must be addressed before any integration can occur.

### 5.1 Interpreting Evidence

Although the conclusions from any study can be misconstrued, fasting and dietary studies are particularly prone to misinterpretation. Specific terminology is often lacking. Fasting, for example, could refer to either a 16-hour TRF or a 7-day prolonged fast, which differ in their impact on metabolism. In another example, “high-fat” diets could refer an LCD or a KD (or neither) depending on the precise amounts of fat and digestible carbohydrate. Specific terminology should be used to describe a metabolic regimen before drawing conclusions about its effects. Moreover, correlative data from observational studies are often used to infer causation. For example, diets high in fat can be associated with adverse cardiovascular outcomes, but this correlation does not permit the inference that the high fat intake was responsible (all potential explanations should be considered, particularly those which acknowledge the deleterious effects of high fat intake virtually always occur in the setting of ample carbohydrate intake) [145]. Causation is best inferred from interventional studies.

### 5.2 Financial Incentives

Financial incentives hinder the adoption of metabolic strategies by healthcare systems on several levels. First, many clinical trials are funded by drug companies and/or conducted by financially conflicted investigators, which is problematic as both practices are associated with favourable outcomes [146,147]. This situation impedes the credibility of many drug trials and deters research into metabolic strategies, for which it is more difficult (though not impossible) to receive financial compensation. Second, clinicians taking part in establishing clinical guidelines are often funded by drug companies [148-150], leading to a conflict of interest. Ideally, investigators and clinicians involved in conducting clinical trials and establishing clinical guidelines should not be financially conflicted. Third, and perhaps the most insidious obstacle, is the mutually beneficial financial incentive that exists between the food and drug industries, with the former promoting food products high in processed carbohydrates, which drive the lifestyle disorders, and the latter promoting drugs that purport to treat the disorders, which prevents patients from recognizing the true problem and altering their food consumption patterns. This obstacle will be challenging to overcome until there is a widespread recognition of the overt harm produced by food products high in processed carbohydrates, which can only occur when government agencies adopt a more decisive stance against these food products in their diet recommendations and guidelines.

### 5.3 Healthcare Education

Clinicians receive virtually no education or training in metabolic strategies. Consequently, many clinicians are not aware of the rationale and evidence underlying fasting and carbohydrate-restricted diets. The current paradigm emphasizes a drug-based approach aimed at suppressing or eliminating disease targets, a model so entrenched that the health-centric notion of restoring mitochondria function is often dismissed outright. This perspective should be challenged early in healthcare education by introducing courses on metabolic strategies into medical school and other health-related curriculums. Moreover, even when motivated, many clinicians lack the experience to guide patients through a fasting or carbohydrate-restricted diet regimen. A potential solution could be to establish “metabolic” fellowship training programs in areas such as endocrinology, cardiology, oncology, and neurology.

### 5.4 Patient Adherence

Patient adherence to metabolic strategies can be low, for two main reasons. First, there is often inadequate support for the strategy. For many patients, the most difficult aspect of a suitable fasting or carbohydrate-restricted diet regimen relates to a lack of understanding and support from society, medical professionals, and even family with respect to its legitimacy and potential health benefits. Without this support, it is difficult for patients to ignore the food industry’s relentless promotion of snacking and processed carbohydrates. Patient education programs can address this issue by educating patients and families on “why” a metabolic strategy may be beneficial. Second, some patients withdraw from a metabolic strategy due to a lack of perceived success or adverse effects. Frustration can arise from an inability to achieve physiological ketosis, which usually results from an inadequate switch to the ketogenic state. Patients can also develop adverse effects, many of which relate to inadequate water or salt intake. Both problems can be promptly addressed with feedback-oriented patient support programs that instruct patients on “how” to follow a metabolic strategy.

## 6.Conclusions

Today’s chosen paradigm of disease and health will have long-lasting repercussions for humanity throughout the remainder of the 21st century. Our current disease-centric paradigm regards the lifestyle disorders as distinct disease processes, caused and driven by specific disease targets that must be suppressed or eliminated to clear the disease, leading to the development of numerous drugs that have yet to halt the rise of these disorders. We may attain more success by employing a health-centric paradigm that recognizes the lifestyle disorders not as disease processes, but as a series of hormonal and metabolic responses to a singular, lifestyle-induced disease of mitochondria dysfunction, a disease target that must be restored to improve health. Dozens of human interventional trials have indicated fasting and carbohydrate-restricted diets can effectively mitigate the metabolic syndrome. Preliminary studies have also indicated potential benefits in atherosclerosis, cancer, and neurodegeneration. Given the existing evidence combined with the ongoing pandemic of disability and death arising from the lifestyle disorders, integrating metabolic strategies into modern healthcare systems should be identified as a global health priority. We now have a golden opportunity to usher in a new era of health. Success lies within our grasp - perhaps it is time to seize it.
